# Crystal structure of hexa­methyl 4,4′,4′′,4′′′,4′′′’,4′′′’’-[(1,3,5,2λ^5^,4λ^5^,6λ^5^-tri­aza­triphosphinine-2,2,4,4,6,6-hexa­yl)hexa­kis­(­oxy)]hexa­benzoate

**DOI:** 10.1107/S2056989017010325

**Published:** 2017-07-25

**Authors:** Jing Zhu, Guo Hui Hou, Qian Li, Fu Wei Zheng, Hong Liang Wei, Hui Juan Chu, Gang Wang, Juan He, Ling Bo Qu

**Affiliations:** a School of Chemical Engineering and Environment, Henan University of Technology, Zhengzhou 450001, People’s Republic of China; b School of Chemistry and Chemical Engineering, Harbin Institute of Technology, Harbin 150001, People’s Republic of China

**Keywords:** crystal structure, cyclo­triphosphazene, organic–inorganic compounds

## Abstract

The title compound, C_48_H_42_N_3_O_18_P_3_, achieved in a two-step synthesis, comprises a cyclo­triphosphazene core and six 4-meth­oxy­carbonyl­phen­oxy groups. Each P atom is attached to two substituents located up and down with respect to the plane of the phosphazene ring, the central P_3_N_3_ ring having a twist-boat conformation. The three O/P/O planes and five of the six benzene rings are nearly perpendicular to the mean plane through the phosphazene ring [dihedral angles = 82.98 (8)–88.92 (8)°], while the remaining benzene ring forms a dihedral angle of 25.72 (7)°. The crystal packing is stabilized by van der Waals inter­actions only.

## Abstract 2

The title compound consists of a cyclo­triphosphazene core and six 4-meth­oxy­carbonyl­phen­oxy groups. The phospho­rus atoms are attached to two substituents located up and down with respect to the plane of the phosphazene ring, the central P_3_N_3_ ring having a twisted-boat conformation.

## Chemical context   

In the past few decades, a rich variety of cyclo­triphosphazenes with inter­esting properties and applications have been synthesized by replacing the Cl atoms of hexa­chloro­cyclo­triphosphazene with various nucleophiles. The properties of cyclo­triphosphazenes depend on the inorganic skeleton, as well as on the nature of the substituents attached to the P atoms (Patil *et al.*, 2011 ▸). Hexa­kis­(allyl 4-hy­droxy­ben­zoate)cyclo­triphosphazene (HABC) possessing six reactive peripheral allyl groups is used as a functional phosphazene-based oligomer for the synthesis of optical resin, through radical homopolymerization of itself and copolymerization with methyl methacrylate (Guo *et al.*, 2009 ▸). The title com­pound, HMPC, was obtained accidentally from the recrystallization of the crude product of HABC. Subsequently, as a retardant additive, HMPC was blended with a polymer of methyl methacrylate to obtain the flame-retardant polymer MC–PMMA. In this context, we report here the synthesis and crystal structure of HMPC.

## Structural commentary   

**Figure 1:**
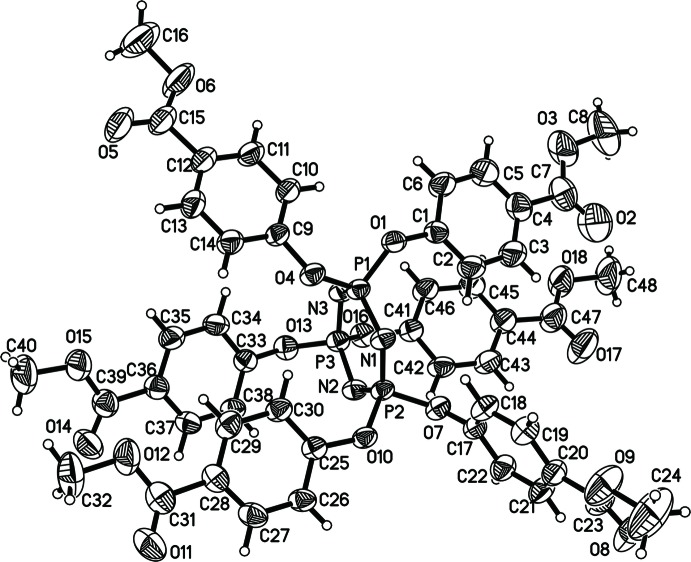
The mol­ecular structure of the title compound showing 50% probability displacement ellipsoids.

The mol­ecule of HMPC (Fig. 1 ▸) comprises a cyclo­triphosphazene core and six 4-meth­oxy­carbonyl phen­oxy groups, and each P atom is attached to two substituents. Three of the six 4-meth­oxy­carbonyl­phen­oxy substituents are on one side of the phosphazene ring, while the other three groups are located on the opposite side. The central phosphazene ring is slightly nonplanar, having a boat distortion, with atoms P1 and N2 lying 0.1223 (7) and 0.138 (2) Å, respectively, on the same side of the plane defined by atoms N1/N3/P2/P3, in agreement with the values reported in the literature for hexa­kis­(4-formyl­phen­oxy)cyclo­triphosphazene (Patil *et al.*, 2011 ▸).

The P—O bond lengths are in the range 1.584 (2)–1.591 (19) Å, with a mean value of 1.584 (2) Å, which is 0.12 Å shorter than the normal single-bond distance (Cruickshank, 1961 ▸), suggesting considerable exocyclic π-bonding. The P—N bond lengths are within a narrow range [1.576 (3)–1.581 (2) Å], indicating electron delocalization within the ring. The N—P—N angles [116.53 (13)–117.92 (12)°] are significantly smaller than the P—N—P angles [121.25 (15)–122.53 (14)°]. The O13—P3—O16 angle [94.40 (12)°] is smaller than the corresponding angles at P1 [99.65 (11)°] and P2 [98.77 (11)°].
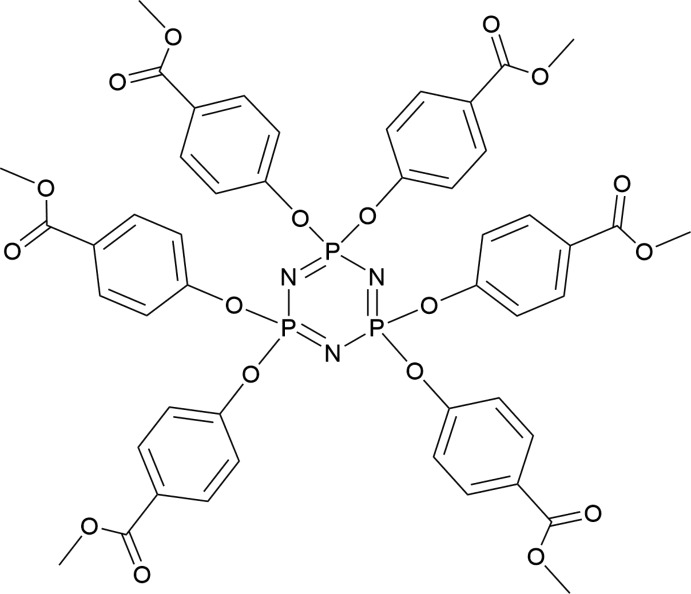


The 4-meth­oxy­carbonyl­phen­oxy groups of the HMPC mol­ecule show significant deviations from a threefold symmetrical arrangement. The three PO_2_ planes (O1/P1/O4, O7/P2/O10 and O13/P3/O16) are nearly perpendicular to the mean plane through the phosphazene ring [dihedral angles = 88.22 (6), 83.79 (9) and 84.84 (6)°, respectively]. Five of the six benzene rings lie approximately perpendicular to the phosphazene ring [dihedral angles = 82.92 (17)–88.16 (13)°; Table 1 ▸], whereas the remaining benzene ring (C17–C22) forms a dihedral angle of 28.21 (14)°. Each benzene ring and its terminal carbonyl group are approximately coplanar, the largest deviation from coplanarity being for the C33–C38 and C36/C39/O14/O15 planes [dihedral angle = 9.96 (14)°].

## Supra­molecular features   

In the title compound, there are no usual hydrogen-bonding or stacking inter­actions, the crystal structure being enforced by van der Waals forces only.

## Database survey   

In a search in the Cambridge Structural Database (Groom *et al.*, 2016 ▸), 15 structures were found incorporating the same cyclo­phosphazene motif substituted by six phen­oxy groups. Of these, only one structure contained alk­oxycarbonyl­phen­oxy groups bonded to each P atom of a phosphazene skeleton (Zhu *et al.*, 2015 ▸). In that structure, the atoms of two terminal propenyl groups are disordered over two sets of sites, with refined site-occupancy ratios of 0.249 (12):0.751 (12) and 0.476 (9):0.524 (9); no inter­molecular inter­actions were observed.

## Synthesis and crystallization   

All of the chemicals and solvents were of reagent grade. Hexa­chloro­cyclo­triphosphazine (HCCP) was purchased from Zhengzhou ALFA Chemical Co. Ltd, recrystallized from dry hexane and sublimated twice. Anhydrous K_2_CO_3_ was activated at 413 K for 2 h. Methyl 4-hy­droxy­benzoate was synthesized according to the literature method of Guo *et al.* (2009 ▸).

A three-necked round-bottomed flask was equipped with a nitro­gen inlet, an addition funnel and a condenser. To a mixture of hexa­chloro­cyclo­triphosphazene (1.04 g, 3 mmol) and anhydrous K_2_CO_3_ (3.5 g, 253 mmol) in tetra­hydro­furan (50 ml), a solution of methyl 4-hy­droxy­benzoate (3.20 g, 21 mmol in tetra­hydro­furan) was added dropwise at room temperature. The reaction mixture was heated at *ca* 338 K for 48 h under nitro­gen and thin-layer chromatography (TLC) was used to monitor the reaction. The resulting suspension was filtered and the filtrate concentrated, leading to the formation of a pale-yellow viscous liquid. This was dissolved in 20 ml ethyl acetate and the solution added dropwise to methanol. Colourless needle-shaped crystals suitable for X-ray diffraction analysis were obtained by slow evaporation of the solvent.

## Refinement   

Crystal data, data collection and structure refinement details are summarized in Table 2 ▸. H atoms were constrained, with C—H = 0.93–0.98 Å and *U*_iso_(H) = 1.5*U*_eq_(C) for methyl H atoms and 1.2*U*_eq_(C) for other H atoms. A rotating model was used for the methyl groups. An ISOR restraint in *SHELXL2014* (Sheldrick, 2015 ▸) was applied to the methyl C16 atom. Ten low-angle reflections with *F*_o_ << *F*_c_, whose intensities may have been significantly reduced by the beam stop, were omitted from the final cycles of refinement.

## Supplementary Material

Crystal structure: contains datablock(s) I. DOI: 10.1107/S2056989017010325/rz5218sup1.cif

Structure factors: contains datablock(s) I. DOI: 10.1107/S2056989017010325/rz5218Isup2.hkl

Supporting information file. DOI: 10.1107/S2056989017010325/rz5218Isup3.cml

CCDC reference: 1561686

Additional supporting information:  crystallographic information; 3D view; checkCIF report

## Figures and Tables

**Table 1:** Dihedral angles between the phosphazene ring and attached benzene rings (°)

Atoms	Angle	Atoms	Angle
C1–C6	84.95 (19)	C25–C30	88.16 (13)
C9–C14	84.12 (16)	C33–C38	83.22 (13)
C17–C22	28.21 (14)	C41–C46	82.92 (17)

**Table 2:** Experimental details

Crystal data	
Chemical formula	C_48_H_42_N_3_O_18_P_3_
*M*_r_	1041.75
Crystal system, space group	Triclinic, *P* 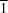
Temperature (K)	291
*a*, *b*, *c* (Å)	11.4012 (4), 13.8443 (5), 17.0264 (8)
α, β, γ (°)	99.134 (3), 95.917 (3), 103.941 (3)
*V* (Å^3^)	2547.07 (18)
*Z*	2
Radiation type	Cu *K*α
μ (mm^−1^)	1.73
Crystal size (mm)	0.2 × 0.18 × 0.16

Data collection	
Diffractometer	Agilent Xcalibur Eos Gemini
Absorption correction	Multi-scan (*CrysAlis PRO*; Agilent, 2014 ▸)
*T*_min_, *T*_max_	0.889, 1.000
No. of measured, independent and observed [*I* > 2σ(*I*)] reflections	18292, 9102, 6523
*R*_int_	0.027
(sin θ/λ)_max_ (Å^−1^)	0.597

Refinement	
*R*[*F* ^2^ > 2σ(*F* ^2^)], *wR*(*F* ^2^), *S*	0.058, 0.181, 1.03
No. of reflections	9102
No. of parameters	654
No. of restraints	6
H-atom treatment	H-atom parameters constrained
Δρ_max_, Δρ_min_ (e Å^−3^)	0.52, −0.28

Computer programs: *CrysAlis PRO* (Agilent, 2014 ▸), *SHELXS97* (Sheldrick, 2008 ▸), *SHELXL2104* (Sheldrick, 2015 ▸) and *OLEX2* (Dolomanov *et al.*, 2009 ▸).

## supplementary crystallographic information

### Crystal data

**Table:**

C_48_H_42_N_3_O_18_P_3_	*Z* = 2
*M**_r_* = 1041.75	*F*(000) = 1080
Triclinic, *P*1	*D*_x_ = 1.358 Mg m^−^^3^
*a* = 11.4012 (4) Å	Cu *K*α radiation, λ = 1.54184 Å
*b* = 13.8443 (5) Å	Cell parameters from 5102 reflections
*c* = 17.0264 (8) Å	θ = 3.9–70.0°
α = 99.134 (3)°	µ = 1.73 mm^−^^1^
β = 95.917 (3)°	*T* = 291 K
γ = 103.941 (3)°	, colourless
*V* = 2547.07 (18) Å^3^	0.2 × 0.18 × 0.16 mm

### Data collection

**Table:**

Agilent Xcalibur Eos Gemini diffractometer	9102 independent reflections
Radiation source: Enhance (Cu) X-ray Source	6523 reflections with *I* > 2σ(*I*)
Graphite monochromator	*R*_int_ = 0.027
Detector resolution: 16.2312 pixels mm^-1^	θ_max_ = 67.1°, θ_min_ = 3.4°
ω scans	*h* = −11→13
Absorption correction: multi-scan (CrysAlis PRO; Agilent, 2014)	*k* = −16→16
*T*_min_ = 0.889, *T*_max_ = 1.000	*l* = −20→20
18292 measured reflections	

### Refinement

**Table:**

Refinement on *F*^2^	Primary atom site location: structure-invariant direct methods
Least-squares matrix: full	Hydrogen site location: mixed
*R*[*F*^2^ > 2σ(*F*^2^)] = 0.058	H-atom parameters constrained
*wR*(*F*^2^) = 0.181	*w* = 1/[σ^2^(*F*_o_^2^) + (0.1121*P*)^2^] where *P* = (*F*_o_^2^ + 2*F*_c_^2^)/3
*S* = 1.03	(Δ/σ)_max_ = 0.001
9102 reflections	Δρ_max_ = 0.52 e Å^−^^3^
654 parameters	Δρ_min_ = −0.28 e Å^−^^3^
6 restraints	

### Special details

**Table:**

Geometry. All esds (except the esd in the dihedral angle between two l.s. planes) are estimated using the full covariance matrix. The cell esds are taken into account individually in the estimation of esds in distances, angles and torsion angles; correlations between esds in cell parameters are only used when they are defined by crystal symmetry. An approximate (isotropic) treatment of cell esds is used for estimating esds involving l.s. planes.

### Fractional atomic coordinates and isotropic or equivalent isotropic displacement parameters (Å^2^)

**Table:**

	*x*	*y*	*z*	*U*_iso_*/*U*_eq_	
C1	0.8359 (3)	0.6168 (2)	0.10677 (19)	0.0668 (7)	
C2	0.8017 (5)	0.6546 (3)	0.1773 (3)	0.1040 (14)	
H2	0.7986	0.7218	0.1886	0.125*	
C3	0.7718 (5)	0.5920 (4)	0.2314 (3)	0.1157 (17)	
H3	0.7484	0.6174	0.2795	0.139*	
C4	0.7758 (4)	0.4932 (3)	0.2158 (3)	0.0933 (11)	
C5	0.8101 (6)	0.4568 (3)	0.1449 (3)	0.1198 (17)	
H5	0.8140	0.3898	0.1337	0.144*	
C6	0.8392 (5)	0.5189 (3)	0.0895 (3)	0.1104 (16)	
H6	0.8607	0.4932	0.0408	0.132*	
C7	0.7458 (6)	0.4277 (5)	0.2764 (4)	0.1261 (18)	
C8	0.7271 (8)	0.2647 (5)	0.3072 (5)	0.194 (4)	
H8A	0.7212	0.1970	0.2802	0.291*	
H8B	0.6515	0.2672	0.3266	0.291*	
H8C	0.7923	0.2843	0.3517	0.291*	
C9	0.8940 (3)	0.7251 (2)	−0.12018 (17)	0.0631 (6)	
C10	0.8708 (4)	0.6214 (3)	−0.1324 (2)	0.0832 (9)	
H10	0.8485	0.5864	−0.0916	0.100*	
C11	0.8814 (5)	0.5699 (3)	−0.2073 (2)	0.0973 (12)	
H11	0.8652	0.4996	−0.2170	0.117*	
C12	0.9155 (4)	0.6220 (3)	−0.2668 (2)	0.0944 (11)	
C13	0.9382 (4)	0.7255 (3)	−0.2527 (2)	0.0983 (12)	
H13	0.9607	0.7607	−0.2933	0.118*	
C14	0.9282 (4)	0.7780 (3)	−0.1791 (2)	0.0837 (10)	
H14	0.9445	0.8484	−0.1695	0.100*	
C15	0.9305 (7)	0.5705 (4)	−0.3476 (3)	0.134 (2)	
C16	0.9187 (9)	0.4122 (6)	−0.4322 (4)	0.214 (4)	
H16A	0.9199	0.4590	−0.4646	0.321*	
H16B	0.8492	0.3557	−0.4450	0.321*	
H16C	0.9920	0.3836	−0.4381	0.321*	
C17	0.8042 (3)	0.9714 (2)	0.26749 (17)	0.0637 (7)	
C18	0.9268 (3)	0.9832 (3)	0.2737 (2)	0.0805 (9)	
H18	0.9599	0.9525	0.2320	0.097*	
C19	1.0025 (4)	1.0417 (3)	0.3428 (2)	0.0868 (10)	
H19	1.0867	1.0514	0.3473	0.104*	
C20	0.9508 (4)	1.0853 (3)	0.4051 (2)	0.0821 (9)	
C21	0.8279 (4)	1.0716 (3)	0.3971 (2)	0.0915 (11)	
H21	0.7940	1.1017	0.4387	0.110*	
C22	0.7527 (4)	1.0146 (3)	0.3292 (2)	0.0828 (9)	
H22	0.6685	1.0052	0.3248	0.099*	
C23	1.0275 (5)	1.1485 (4)	0.4813 (3)	0.1073 (14)	
C24	1.2295 (7)	1.2371 (7)	0.5482 (4)	0.222 (4)	
H24A	1.3126	1.2378	0.5426	0.333*	
H24B	1.2090	1.2113	0.5956	0.333*	
H24C	1.2202	1.3048	0.5529	0.333*	
C25	0.7969 (3)	1.0760 (2)	0.04436 (18)	0.0621 (6)	
C26	0.7774 (3)	1.1707 (2)	0.0538 (2)	0.0733 (8)	
H26	0.7537	1.1983	0.1011	0.088*	
C27	0.7933 (3)	1.2244 (2)	−0.0070 (2)	0.0814 (9)	
H27	0.7801	1.2888	−0.0006	0.098*	
C28	0.8281 (3)	1.1854 (3)	−0.0771 (2)	0.0756 (8)	
C29	0.8469 (3)	1.0887 (3)	−0.0863 (2)	0.0796 (9)	
H29	0.8695	1.0609	−0.1340	0.096*	
C30	0.8322 (3)	1.0334 (2)	−0.0248 (2)	0.0725 (8)	
H30	0.8460	0.9693	−0.0305	0.087*	
C31	0.8456 (4)	1.2499 (3)	−0.1411 (3)	0.0929 (11)	
C32	0.8839 (7)	1.2588 (5)	−0.2720 (3)	0.161 (3)	
H32A	0.9126	1.2234	−0.3161	0.242*	
H32B	0.9409	1.3236	−0.2518	0.242*	
H32C	0.8056	1.2683	−0.2902	0.242*	
C33	0.5371 (3)	0.8246 (2)	−0.12920 (19)	0.0662 (7)	
C34	0.6005 (4)	0.7933 (3)	−0.1888 (2)	0.0815 (9)	
H34	0.6208	0.7318	−0.1920	0.098*	
C35	0.6331 (4)	0.8545 (3)	−0.2430 (2)	0.0853 (10)	
H35	0.6774	0.8350	−0.2827	0.102*	
C36	0.6008 (3)	0.9452 (2)	−0.2394 (2)	0.0738 (8)	
C37	0.5407 (3)	0.9764 (2)	−0.1778 (2)	0.0746 (8)	
H37	0.5207	1.0380	−0.1742	0.089*	
C38	0.5102 (3)	0.9169 (2)	−0.1216 (2)	0.0709 (7)	
H38	0.4718	0.9389	−0.0792	0.085*	
C39	0.6214 (4)	1.0076 (3)	−0.3021 (3)	0.0902 (10)	
C40	0.6989 (7)	1.0189 (6)	−0.4250 (4)	0.170 (3)	
H40A	0.6492	0.9724	−0.4710	0.254*	
H40B	0.7827	1.0350	−0.4338	0.254*	
H40C	0.6719	1.0798	−0.4168	0.254*	
C41	0.4629 (3)	0.6495 (2)	0.1052 (2)	0.0692 (7)	
C42	0.4727 (4)	0.7170 (2)	0.1749 (2)	0.0861 (10)	
H42	0.4812	0.7854	0.1743	0.103*	
C43	0.4698 (4)	0.6821 (3)	0.2460 (3)	0.0963 (12)	
H43	0.4778	0.7277	0.2939	0.116*	
C44	0.4553 (4)	0.5804 (3)	0.2475 (3)	0.0927 (11)	
C45	0.4436 (5)	0.5145 (3)	0.1758 (3)	0.1006 (13)	
H45	0.4327	0.4458	0.1759	0.121*	
C46	0.4477 (4)	0.5485 (2)	0.1038 (2)	0.0882 (10)	
H46	0.4402	0.5036	0.0556	0.106*	
C47	0.4519 (6)	0.5466 (4)	0.3263 (3)	0.1229 (17)	
C48	0.4132 (10)	0.4064 (5)	0.3942 (4)	0.212 (4)	
H48A	0.3850	0.3338	0.3812	0.317*	
H48B	0.3574	0.4330	0.4242	0.317*	
H48C	0.4928	0.4259	0.4258	0.317*	
N1	0.8413 (2)	0.85679 (17)	0.08693 (14)	0.0609 (5)	
N2	0.6062 (2)	0.86386 (17)	0.05940 (15)	0.0623 (5)	
N3	0.6736 (2)	0.70238 (16)	−0.01228 (15)	0.0640 (6)	
O1	0.87654 (19)	0.67662 (15)	0.05182 (13)	0.0678 (5)	
O2	0.7203 (6)	0.4592 (4)	0.3418 (3)	0.200 (3)	
O3	0.7516 (4)	0.3338 (3)	0.2515 (3)	0.1446 (15)	
O4	0.89006 (19)	0.78510 (14)	−0.04713 (12)	0.0672 (5)	
O5	0.9558 (5)	0.6131 (3)	−0.4018 (2)	0.175 (2)	
O6	0.9121 (7)	0.4724 (3)	−0.3521 (2)	0.212 (3)	
O7	0.72241 (19)	0.91374 (15)	0.20060 (12)	0.0689 (5)	
O8	0.9856 (4)	1.1792 (3)	0.53921 (19)	0.1426 (14)	
O9	1.1461 (4)	1.1706 (3)	0.4758 (2)	0.1500 (16)	
O10	0.78245 (19)	1.02854 (13)	0.11051 (12)	0.0656 (5)	
O11	0.8401 (3)	1.3358 (2)	−0.1305 (2)	0.1172 (11)	
O12	0.8724 (3)	1.1990 (3)	−0.20758 (19)	0.1173 (10)	
O13	0.49148 (19)	0.75886 (16)	−0.07913 (14)	0.0731 (5)	
O14	0.5805 (3)	1.0779 (3)	−0.3076 (2)	0.1237 (12)	
O15	0.6883 (3)	0.9731 (3)	−0.35497 (19)	0.1156 (10)	
O16	0.45734 (19)	0.68050 (16)	0.03086 (14)	0.0735 (5)	
O17	0.4720 (6)	0.6033 (3)	0.3903 (2)	0.197 (3)	
O18	0.4200 (4)	0.4473 (3)	0.3187 (2)	0.1383 (14)	
P1	0.81205 (6)	0.75616 (5)	0.02155 (4)	0.05769 (19)	
P2	0.73803 (6)	0.90996 (5)	0.10884 (4)	0.05735 (19)	
P3	0.56765 (6)	0.75527 (5)	0.00331 (5)	0.0604 (2)	

### Atomic displacement parameters (Å^2^)

**Table:**

	*U*^11^	*U*^22^	*U*^33^	*U*^12^	*U*^13^	*U*^23^
C1	0.0705 (17)	0.0575 (15)	0.0731 (18)	0.0171 (13)	0.0139 (13)	0.0119 (13)
C2	0.163 (4)	0.075 (2)	0.098 (3)	0.051 (2)	0.058 (3)	0.030 (2)
C3	0.176 (5)	0.101 (3)	0.096 (3)	0.058 (3)	0.062 (3)	0.034 (2)
C4	0.101 (3)	0.079 (2)	0.099 (3)	0.0106 (19)	0.016 (2)	0.033 (2)
C5	0.197 (5)	0.0553 (19)	0.106 (3)	0.025 (3)	0.028 (3)	0.020 (2)
C6	0.193 (5)	0.0594 (19)	0.084 (2)	0.037 (2)	0.040 (3)	0.0129 (17)
C7	0.136 (4)	0.126 (4)	0.127 (4)	0.024 (3)	0.030 (3)	0.066 (4)
C8	0.225 (8)	0.137 (5)	0.209 (7)	−0.011 (5)	−0.007 (6)	0.117 (5)
C9	0.0649 (16)	0.0627 (15)	0.0627 (15)	0.0167 (12)	0.0199 (12)	0.0080 (12)
C10	0.117 (3)	0.0666 (18)	0.0684 (19)	0.0225 (18)	0.0316 (18)	0.0120 (15)
C11	0.152 (4)	0.0656 (19)	0.081 (2)	0.032 (2)	0.045 (2)	0.0087 (17)
C12	0.131 (3)	0.089 (2)	0.072 (2)	0.038 (2)	0.038 (2)	0.0095 (18)
C13	0.139 (4)	0.092 (3)	0.074 (2)	0.034 (2)	0.045 (2)	0.0244 (19)
C14	0.107 (3)	0.0666 (18)	0.084 (2)	0.0230 (17)	0.040 (2)	0.0178 (16)
C15	0.227 (7)	0.106 (3)	0.088 (3)	0.061 (4)	0.074 (4)	0.016 (3)
C16	0.328 (8)	0.169 (5)	0.155 (5)	0.083 (5)	0.110 (5)	−0.014 (4)
C17	0.0808 (18)	0.0504 (13)	0.0599 (15)	0.0099 (12)	0.0247 (13)	0.0119 (11)
C18	0.088 (2)	0.081 (2)	0.0686 (18)	0.0204 (17)	0.0250 (16)	−0.0036 (16)
C19	0.091 (2)	0.088 (2)	0.075 (2)	0.0173 (18)	0.0147 (17)	0.0037 (18)
C20	0.113 (3)	0.0716 (19)	0.0609 (17)	0.0218 (18)	0.0194 (17)	0.0089 (14)
C21	0.120 (3)	0.097 (3)	0.0629 (19)	0.037 (2)	0.0337 (19)	0.0036 (17)
C22	0.093 (2)	0.090 (2)	0.0704 (19)	0.0284 (18)	0.0293 (17)	0.0135 (17)
C23	0.142 (4)	0.092 (3)	0.076 (2)	0.018 (3)	0.016 (3)	−0.001 (2)
C24	0.183 (7)	0.250 (10)	0.144 (6)	−0.014 (7)	−0.034 (5)	−0.079 (6)
C25	0.0599 (15)	0.0486 (13)	0.0720 (17)	0.0011 (11)	0.0186 (12)	0.0094 (12)
C26	0.0802 (19)	0.0521 (15)	0.090 (2)	0.0130 (13)	0.0311 (16)	0.0136 (14)
C27	0.083 (2)	0.0612 (17)	0.106 (3)	0.0174 (15)	0.0278 (19)	0.0248 (17)
C28	0.0622 (17)	0.0729 (19)	0.091 (2)	0.0077 (14)	0.0139 (15)	0.0277 (17)
C29	0.079 (2)	0.079 (2)	0.075 (2)	0.0061 (16)	0.0244 (16)	0.0116 (16)
C30	0.084 (2)	0.0537 (15)	0.0767 (19)	0.0086 (13)	0.0284 (16)	0.0075 (13)
C31	0.073 (2)	0.094 (3)	0.112 (3)	0.0093 (18)	0.022 (2)	0.038 (2)
C32	0.192 (7)	0.183 (6)	0.112 (4)	0.018 (5)	0.030 (4)	0.081 (4)
C33	0.0641 (16)	0.0580 (15)	0.0710 (17)	0.0091 (12)	0.0065 (13)	0.0092 (13)
C34	0.099 (2)	0.0618 (17)	0.093 (2)	0.0309 (16)	0.0268 (19)	0.0159 (16)
C35	0.100 (3)	0.081 (2)	0.086 (2)	0.0351 (19)	0.038 (2)	0.0176 (18)
C36	0.0721 (18)	0.0702 (18)	0.082 (2)	0.0176 (14)	0.0183 (15)	0.0195 (15)
C37	0.0750 (19)	0.0611 (16)	0.093 (2)	0.0214 (14)	0.0203 (16)	0.0191 (15)
C38	0.0690 (17)	0.0643 (17)	0.0809 (19)	0.0183 (13)	0.0215 (15)	0.0104 (14)
C39	0.090 (2)	0.094 (3)	0.096 (3)	0.025 (2)	0.026 (2)	0.031 (2)
C40	0.214 (7)	0.221 (7)	0.137 (5)	0.102 (6)	0.097 (5)	0.104 (5)
C41	0.0680 (17)	0.0570 (15)	0.0798 (19)	0.0053 (12)	0.0246 (14)	0.0132 (14)
C42	0.109 (3)	0.0528 (16)	0.100 (3)	0.0181 (16)	0.044 (2)	0.0126 (16)
C43	0.127 (3)	0.071 (2)	0.089 (2)	0.017 (2)	0.044 (2)	0.0059 (18)
C44	0.114 (3)	0.070 (2)	0.090 (2)	0.0083 (19)	0.033 (2)	0.0135 (18)
C45	0.144 (4)	0.0541 (17)	0.098 (3)	0.0048 (19)	0.033 (3)	0.0176 (18)
C46	0.119 (3)	0.0511 (16)	0.085 (2)	0.0026 (16)	0.030 (2)	0.0061 (15)
C47	0.175 (5)	0.096 (3)	0.095 (3)	0.017 (3)	0.041 (3)	0.023 (3)
C48	0.386 (14)	0.140 (6)	0.104 (4)	0.041 (7)	0.035 (6)	0.053 (4)
N1	0.0613 (13)	0.0505 (11)	0.0661 (13)	0.0092 (9)	0.0160 (10)	0.0008 (10)
N2	0.0612 (13)	0.0499 (11)	0.0745 (14)	0.0119 (9)	0.0187 (11)	0.0063 (10)
N3	0.0686 (14)	0.0436 (10)	0.0733 (14)	0.0076 (9)	0.0146 (11)	0.0002 (10)
O1	0.0766 (12)	0.0607 (11)	0.0730 (12)	0.0249 (9)	0.0250 (10)	0.0135 (9)
O2	0.296 (7)	0.189 (5)	0.171 (4)	0.082 (5)	0.116 (5)	0.114 (4)
O3	0.188 (4)	0.090 (2)	0.150 (3)	0.006 (2)	0.009 (3)	0.062 (2)
O4	0.0779 (12)	0.0522 (10)	0.0688 (12)	0.0100 (9)	0.0254 (10)	0.0053 (9)
O5	0.322 (7)	0.128 (3)	0.095 (2)	0.068 (4)	0.103 (3)	0.022 (2)
O6	0.443 (9)	0.108 (3)	0.113 (3)	0.089 (4)	0.143 (4)	0.014 (2)
O7	0.0771 (12)	0.0576 (10)	0.0671 (12)	0.0039 (9)	0.0276 (10)	0.0077 (9)
O8	0.185 (4)	0.153 (3)	0.0691 (18)	0.024 (3)	0.029 (2)	−0.0143 (19)
O9	0.129 (3)	0.162 (4)	0.111 (3)	0.004 (3)	−0.010 (2)	−0.044 (2)
O10	0.0823 (13)	0.0441 (9)	0.0663 (11)	0.0078 (8)	0.0231 (9)	0.0036 (8)
O11	0.123 (2)	0.101 (2)	0.149 (3)	0.0353 (18)	0.037 (2)	0.064 (2)
O12	0.136 (3)	0.126 (3)	0.093 (2)	0.018 (2)	0.0320 (19)	0.0453 (19)
O13	0.0694 (12)	0.0624 (11)	0.0809 (14)	0.0041 (9)	0.0096 (10)	0.0150 (10)
O14	0.145 (3)	0.117 (2)	0.149 (3)	0.063 (2)	0.061 (2)	0.073 (2)
O15	0.139 (3)	0.130 (2)	0.106 (2)	0.052 (2)	0.057 (2)	0.0526 (19)
O16	0.0669 (12)	0.0598 (11)	0.0865 (14)	−0.0005 (9)	0.0163 (10)	0.0150 (10)
O17	0.366 (8)	0.116 (3)	0.096 (3)	0.025 (4)	0.074 (4)	0.014 (2)
O18	0.222 (4)	0.090 (2)	0.093 (2)	0.011 (2)	0.031 (2)	0.0313 (17)
P1	0.0640 (4)	0.0458 (3)	0.0621 (4)	0.0113 (3)	0.0187 (3)	0.0054 (3)
P2	0.0641 (4)	0.0429 (3)	0.0623 (4)	0.0068 (3)	0.0215 (3)	0.0051 (3)
P3	0.0596 (4)	0.0465 (3)	0.0706 (4)	0.0050 (3)	0.0146 (3)	0.0083 (3)

### Geometric parameters (Å, º)

**Table:**

C1—C2	1.366 (5)	C27—C28	1.367 (5)
C1—C6	1.351 (5)	C28—C29	1.392 (5)
C1—O1	1.388 (4)	C28—C31	1.514 (5)
C2—H2	0.9300	C29—H29	0.9300
C2—C3	1.375 (5)	C29—C30	1.393 (5)
C3—H3	0.9300	C30—H30	0.9300
C3—C4	1.364 (6)	C31—O11	1.193 (5)
C4—C5	1.364 (6)	C31—O12	1.339 (5)
C4—C7	1.492 (6)	C32—H32A	0.9600
C5—H5	0.9300	C32—H32B	0.9600
C5—C6	1.387 (6)	C32—H32C	0.9600
C6—H6	0.9300	C32—O12	1.474 (5)
C7—O2	1.220 (7)	C33—C34	1.379 (5)
C7—O3	1.322 (7)	C33—C38	1.374 (4)
C8—H8A	0.9600	C33—O13	1.391 (4)
C8—H8B	0.9600	C34—H34	0.9300
C8—H8C	0.9600	C34—C35	1.370 (5)
C8—O3	1.451 (6)	C35—H35	0.9300
C9—C10	1.373 (4)	C35—C36	1.384 (5)
C9—C14	1.368 (4)	C36—C37	1.379 (5)
C9—O4	1.393 (3)	C36—C39	1.479 (5)
C10—H10	0.9300	C37—H37	0.9300
C10—C11	1.390 (5)	C37—C38	1.376 (5)
C11—H11	0.9300	C38—H38	0.9300
C11—C12	1.370 (5)	C39—O14	1.189 (5)
C12—C13	1.371 (5)	C39—O15	1.342 (5)
C12—C15	1.493 (5)	C40—H40A	0.9600
C13—H13	0.9300	C40—H40B	0.9600
C13—C14	1.376 (5)	C40—H40C	0.9600
C14—H14	0.9300	C40—O15	1.439 (5)
C15—O5	1.194 (6)	C41—C42	1.366 (5)
C15—O6	1.312 (6)	C41—C46	1.363 (4)
C16—H16A	0.9140	C41—O16	1.400 (4)
C16—H16B	0.9484	C42—H42	0.9300
C16—H16C	1.0149	C42—C43	1.372 (5)
C16—O6	1.500 (6)	C43—H43	0.9300
C17—C18	1.359 (5)	C43—C44	1.383 (5)
C17—C22	1.379 (4)	C44—C45	1.375 (5)
C17—O7	1.392 (4)	C44—C47	1.491 (6)
C18—H18	0.9300	C45—H45	0.9300
C18—C19	1.392 (5)	C45—C46	1.383 (5)
C19—H19	0.9300	C46—H46	0.9300
C19—C20	1.391 (5)	C47—O17	1.203 (6)
C20—C21	1.357 (6)	C47—O18	1.316 (6)
C20—C23	1.499 (6)	C48—H48A	0.9600
C21—H21	0.9300	C48—H48B	0.9600
C21—C22	1.369 (5)	C48—H48C	0.9600
C22—H22	0.9300	C48—O18	1.485 (6)
C23—O8	1.202 (5)	N1—P1	1.581 (2)
C23—O9	1.330 (6)	N1—P2	1.578 (2)
C24—H24A	0.9600	N2—P2	1.576 (3)
C24—H24B	0.9600	N2—P3	1.583 (2)
C24—H24C	0.9600	N3—P1	1.578 (2)
C24—O9	1.500 (6)	N3—P3	1.580 (2)
C25—C26	1.369 (4)	O1—P1	1.585 (2)
C25—C30	1.375 (4)	O4—P1	1.584 (2)
C25—O10	1.396 (3)	O7—P2	1.585 (2)
C26—H26	0.9300	O10—P2	1.5919 (19)
C26—C27	1.371 (5)	O13—P3	1.587 (2)
C27—H27	0.9300	O16—P3	1.588 (2)
			
C2—C1—O1	122.8 (3)	C29—C30—H30	120.9
C6—C1—C2	120.8 (3)	O11—C31—C28	122.8 (4)
C6—C1—O1	116.3 (3)	O11—C31—O12	126.0 (4)
C1—C2—H2	120.4	O12—C31—C28	111.1 (4)
C1—C2—C3	119.1 (4)	H32A—C32—H32B	109.5
C3—C2—H2	120.4	H32A—C32—H32C	109.5
C2—C3—H3	119.4	H32B—C32—H32C	109.5
C4—C3—C2	121.2 (4)	O12—C32—H32A	109.5
C4—C3—H3	119.4	O12—C32—H32B	109.5
C3—C4—C5	118.7 (4)	O12—C32—H32C	109.5
C3—C4—C7	120.0 (5)	C34—C33—O13	119.6 (3)
C5—C4—C7	121.2 (4)	C38—C33—C34	121.4 (3)
C4—C5—H5	119.7	C38—C33—O13	118.8 (3)
C4—C5—C6	120.6 (4)	C33—C34—H34	120.6
C6—C5—H5	119.7	C35—C34—C33	118.7 (3)
C1—C6—C5	119.5 (4)	C35—C34—H34	120.6
C1—C6—H6	120.2	C34—C35—H35	119.6
C5—C6—H6	120.2	C34—C35—C36	120.8 (3)
O2—C7—C4	122.8 (6)	C36—C35—H35	119.6
O2—C7—O3	124.6 (5)	C35—C36—C39	122.5 (3)
O3—C7—C4	112.5 (5)	C37—C36—C35	119.3 (3)
H8A—C8—H8B	109.5	C37—C36—C39	118.1 (3)
H8A—C8—H8C	109.5	C36—C37—H37	119.8
H8B—C8—H8C	109.5	C38—C37—C36	120.5 (3)
O3—C8—H8A	109.5	C38—C37—H37	119.8
O3—C8—H8B	109.5	C33—C38—C37	119.1 (3)
O3—C8—H8C	109.5	C33—C38—H38	120.5
C10—C9—O4	123.6 (3)	C37—C38—H38	120.5
C14—C9—C10	121.7 (3)	O14—C39—C36	124.8 (4)
C14—C9—O4	114.5 (3)	O14—C39—O15	123.2 (4)
C9—C10—H10	120.8	O15—C39—C36	112.0 (3)
C9—C10—C11	118.4 (3)	H40A—C40—H40B	109.5
C11—C10—H10	120.8	H40A—C40—H40C	109.5
C10—C11—H11	119.8	H40B—C40—H40C	109.5
C12—C11—C10	120.5 (3)	O15—C40—H40A	109.5
C12—C11—H11	119.8	O15—C40—H40B	109.5
C11—C12—C13	119.8 (3)	O15—C40—H40C	109.5
C11—C12—C15	122.6 (4)	C42—C41—O16	120.7 (3)
C13—C12—C15	117.6 (4)	C46—C41—C42	122.1 (3)
C12—C13—H13	119.6	C46—C41—O16	116.9 (3)
C12—C13—C14	120.7 (3)	C41—C42—H42	120.6
C14—C13—H13	119.6	C41—C42—C43	118.8 (3)
C9—C14—C13	118.9 (3)	C43—C42—H42	120.6
C9—C14—H14	120.6	C42—C43—H43	119.5
C13—C14—H14	120.6	C42—C43—C44	121.1 (4)
O5—C15—C12	124.3 (5)	C44—C43—H43	119.5
O5—C15—O6	124.0 (4)	C43—C44—C47	118.8 (4)
O6—C15—C12	111.6 (4)	C45—C44—C43	118.4 (4)
H16A—C16—H16B	114.6	C45—C44—C47	122.8 (4)
H16A—C16—H16C	108.6	C44—C45—H45	119.3
H16B—C16—H16C	105.9	C44—C45—C46	121.3 (3)
O6—C16—H16A	101.8	C46—C45—H45	119.3
O6—C16—H16B	107.9	C41—C46—C45	118.3 (3)
O6—C16—H16C	118.4	C41—C46—H46	120.9
C18—C17—C22	121.2 (3)	C45—C46—H46	120.9
C18—C17—O7	123.0 (3)	O17—C47—C44	124.1 (5)
C22—C17—O7	115.7 (3)	O17—C47—O18	122.9 (5)
C17—C18—H18	120.2	O18—C47—C44	112.9 (4)
C17—C18—C19	119.6 (3)	H48A—C48—H48B	109.5
C19—C18—H18	120.2	H48A—C48—H48C	109.5
C18—C19—H19	120.4	H48B—C48—H48C	109.5
C20—C19—C18	119.2 (4)	O18—C48—H48A	109.5
C20—C19—H19	120.4	O18—C48—H48B	109.5
C19—C20—C23	121.7 (4)	O18—C48—H48C	109.5
C21—C20—C19	119.6 (4)	P2—N1—P1	121.25 (15)
C21—C20—C23	118.7 (4)	P2—N2—P3	122.18 (15)
C20—C21—H21	119.2	P1—N3—P3	122.53 (14)
C20—C21—C22	121.6 (3)	C1—O1—P1	125.06 (18)
C22—C21—H21	119.2	C7—O3—C8	116.7 (5)
C17—C22—H22	120.6	C9—O4—P1	128.66 (18)
C21—C22—C17	118.7 (4)	C15—O6—C16	116.2 (5)
C21—C22—H22	120.6	C17—O7—P2	127.46 (17)
O8—C23—C20	123.5 (5)	C23—O9—C24	116.0 (5)
O8—C23—O9	124.1 (5)	C25—O10—P2	126.87 (17)
O9—C23—C20	112.3 (4)	C31—O12—C32	111.9 (4)
H24A—C24—H24B	109.5	C33—O13—P3	122.39 (19)
H24A—C24—H24C	109.5	C39—O15—C40	116.6 (4)
H24B—C24—H24C	109.5	C41—O16—P3	124.1 (2)
O9—C24—H24A	109.5	C47—O18—C48	116.8 (4)
O9—C24—H24B	109.5	N1—P1—O1	110.93 (13)
O9—C24—H24C	109.5	N1—P1—O4	105.08 (11)
C26—C25—C30	121.5 (3)	N3—P1—N1	117.61 (12)
C26—C25—O10	115.3 (3)	N3—P1—O1	109.56 (12)
C30—C25—O10	123.2 (3)	N3—P1—O4	112.49 (13)
C25—C26—H26	120.3	O4—P1—O1	99.65 (11)
C25—C26—C27	119.4 (3)	N1—P2—O7	110.81 (13)
C27—C26—H26	120.3	N1—P2—O10	110.56 (12)
C26—C27—H27	119.3	N2—P2—N1	117.92 (12)
C28—C27—C26	121.3 (3)	N2—P2—O7	106.22 (12)
C28—C27—H27	119.3	N2—P2—O10	110.78 (12)
C27—C28—C29	118.8 (3)	O7—P2—O10	98.77 (11)
C27—C28—C31	118.3 (3)	N2—P3—O13	110.06 (13)
C29—C28—C31	122.9 (4)	N2—P3—O16	112.16 (12)
C28—C29—H29	119.7	N3—P3—N2	116.53 (13)
C28—C29—C30	120.7 (3)	N3—P3—O13	110.80 (13)
C30—C29—H29	119.7	N3—P3—O16	110.73 (12)
C25—C30—C29	118.3 (3)	O13—P3—O16	94.40 (12)
C25—C30—H30	120.9		
			
C1—C2—C3—C4	0.0 (8)	C33—C34—C35—C36	−1.4 (6)
C1—O1—P1—N1	77.4 (3)	C33—O13—P3—N2	56.8 (3)
C1—O1—P1—N3	−54.1 (3)	C33—O13—P3—N3	−73.5 (3)
C1—O1—P1—O4	−172.2 (2)	C33—O13—P3—O16	172.4 (2)
C2—C1—C6—C5	−1.5 (8)	C34—C33—C38—C37	4.0 (5)
C2—C1—O1—P1	−46.9 (5)	C34—C33—O13—P3	90.7 (3)
C2—C3—C4—C5	−0.1 (9)	C34—C35—C36—C37	3.4 (6)
C2—C3—C4—C7	178.5 (5)	C34—C35—C36—C39	−172.7 (4)
C3—C4—C5—C6	−0.6 (8)	C35—C36—C37—C38	−1.7 (6)
C3—C4—C7—O2	−2.1 (10)	C35—C36—C39—O14	169.1 (5)
C3—C4—C7—O3	178.8 (5)	C35—C36—C39—O15	−8.4 (6)
C4—C5—C6—C1	1.4 (9)	C36—C37—C38—C33	−2.0 (5)
C4—C7—O3—C8	178.4 (5)	C36—C39—O15—C40	171.3 (5)
C5—C4—C7—O2	176.5 (7)	C37—C36—C39—O14	−7.1 (7)
C5—C4—C7—O3	−2.6 (8)	C37—C36—C39—O15	175.5 (4)
C6—C1—C2—C3	0.8 (7)	C38—C33—C34—C35	−2.4 (6)
C6—C1—O1—P1	137.2 (3)	C38—C33—O13—P3	−93.9 (3)
C7—C4—C5—C6	−179.2 (5)	C39—C36—C37—C38	174.6 (3)
C9—C10—C11—C12	0.7 (7)	C41—C42—C43—C44	−1.0 (7)
C9—O4—P1—N1	−177.2 (2)	C41—O16—P3—N2	−62.6 (3)
C9—O4—P1—N3	−48.1 (3)	C41—O16—P3—N3	69.4 (3)
C9—O4—P1—O1	67.9 (3)	C41—O16—P3—O13	−176.4 (2)
C10—C9—C14—C13	0.7 (6)	C42—C41—C46—C45	−0.7 (6)
C10—C9—O4—P1	−27.7 (4)	C42—C41—O16—P3	67.6 (4)
C10—C11—C12—C13	−0.6 (8)	C42—C43—C44—C45	−0.1 (7)
C10—C11—C12—C15	178.8 (5)	C42—C43—C44—C47	−179.6 (5)
C11—C12—C13—C14	0.6 (8)	C43—C44—C45—C46	0.8 (7)
C11—C12—C15—O5	177.2 (7)	C43—C44—C47—O17	−7.0 (10)
C11—C12—C15—O6	−2.7 (9)	C43—C44—C47—O18	170.6 (5)
C12—C13—C14—C9	−0.6 (7)	C44—C45—C46—C41	−0.4 (7)
C12—C15—O6—C16	177.1 (6)	C44—C47—O18—C48	−179.6 (6)
C13—C12—C15—O5	−3.3 (10)	C45—C44—C47—O17	173.6 (7)
C13—C12—C15—O6	176.8 (6)	C45—C44—C47—O18	−8.8 (8)
C14—C9—C10—C11	−0.8 (6)	C46—C41—C42—C43	1.4 (6)
C14—C9—O4—P1	155.0 (3)	C46—C41—O16—P3	−117.7 (3)
C15—C12—C13—C14	−178.9 (5)	C47—C44—C45—C46	−179.7 (5)
C17—C18—C19—C20	1.3 (6)	O1—C1—C2—C3	−174.9 (4)
C17—O7—P2—N1	69.3 (3)	O1—C1—C6—C5	174.4 (4)
C17—O7—P2—N2	−161.5 (2)	O2—C7—O3—C8	−0.7 (10)
C17—O7—P2—O10	−46.7 (3)	O4—C9—C10—C11	−177.9 (4)
C18—C17—C22—C21	1.0 (5)	O4—C9—C14—C13	178.1 (4)
C18—C17—O7—P2	−42.0 (4)	O5—C15—O6—C16	−2.8 (13)
C18—C19—C20—C21	−1.0 (6)	O7—C17—C18—C19	−179.6 (3)
C18—C19—C20—C23	179.6 (4)	O7—C17—C22—C21	179.5 (3)
C19—C20—C21—C22	0.8 (6)	O8—C23—O9—C24	0.9 (9)
C19—C20—C23—O8	−172.6 (5)	O10—C25—C26—C27	178.0 (3)
C19—C20—C23—O9	10.5 (6)	O10—C25—C30—C29	−178.3 (3)
C20—C21—C22—C17	−0.8 (6)	O11—C31—O12—C32	−5.5 (7)
C20—C23—O9—C24	177.7 (5)	O13—C33—C34—C35	172.9 (3)
C21—C20—C23—O8	8.1 (7)	O13—C33—C38—C37	−171.3 (3)
C21—C20—C23—O9	−168.8 (4)	O14—C39—O15—C40	−6.3 (8)
C22—C17—C18—C19	−1.3 (5)	O16—C41—C42—C43	175.8 (3)
C22—C17—O7—P2	139.6 (3)	O16—C41—C46—C45	−175.3 (4)
C23—C20—C21—C22	−179.8 (4)	O17—C47—O18—C48	−1.9 (11)
C25—C26—C27—C28	0.0 (5)	P1—N1—P2—N2	−4.0 (2)
C25—O10—P2—N1	72.8 (3)	P1—N1—P2—O7	118.61 (17)
C25—O10—P2—N2	−59.8 (3)	P1—N1—P2—O10	−132.93 (16)
C25—O10—P2—O7	−171.0 (2)	P1—N3—P3—N2	−3.4 (2)
C26—C25—C30—C29	−0.6 (5)	P1—N3—P3—O13	123.41 (18)
C26—C25—O10—P2	149.5 (2)	P1—N3—P3—O16	−133.21 (18)
C26—C27—C28—C29	0.4 (5)	P2—N1—P1—N3	−8.3 (2)
C26—C27—C28—C31	−179.1 (3)	P2—N1—P1—O1	−135.53 (16)
C27—C28—C29—C30	−0.9 (5)	P2—N1—P1—O4	117.68 (17)
C27—C28—C31—O11	6.3 (6)	P2—N2—P3—N3	−9.8 (2)
C27—C28—C31—O12	−176.3 (3)	P2—N2—P3—O13	−137.04 (17)
C28—C29—C30—C25	1.0 (5)	P2—N2—P3—O16	119.26 (17)
C28—C31—O12—C32	177.2 (4)	P3—N2—P2—N1	13.5 (2)
C29—C28—C31—O11	−173.1 (4)	P3—N2—P2—O7	−111.43 (18)
C29—C28—C31—O12	4.2 (5)	P3—N2—P2—O10	142.30 (16)
C30—C25—C26—C27	0.1 (5)	P3—N3—P1—N1	12.3 (3)
C30—C25—O10—P2	−32.7 (4)	P3—N3—P1—O1	140.13 (17)
C31—C28—C29—C30	178.6 (3)	P3—N3—P1—O4	−110.02 (18)
